# Novel hub genes associated with pulmonary artery remodeling in pulmonary hypertension

**DOI:** 10.3389/fcvm.2022.945854

**Published:** 2022-11-30

**Authors:** Rubin Tan, Qiang You, Dongdong Yu, Chushu Xiao, Joseph Adu-Amankwaah, Jie Cui, Ting Zhang

**Affiliations:** ^1^Department of Physiology, Basic Medical School, Xuzhou Medical University, Xuzhou, China; ^2^School of Pharmacy, Shandong University of Traditional Chinese Medicine, Jinan, Shandong, China; ^3^Department of Tumor Radiotherapy, Renmin Hospital of Wuhan University, Wuhan, China; ^4^Department of Pathophysiology, School of Basic Medicine, Huazhong University of Science and Technology, Wuhan, China; ^5^Key Laboratory of Pulmonary Diseases of Ministry of Health, Tongji Medical College, Huazhong University of Science and Technology, Wuhan, China

**Keywords:** pulmonary hypertension, idiopathic pulmonary artery hypertension, chronic thromboembolic pulmonary hypertension, pulmonary artery remodeling, bioinformatic analysis

## Abstract

Pulmonary hypertension (PH) is a life-threatening disease with complex pathogenesis. According to etiology, PH is divided into five major groups in clinical classification. However, pulmonary artery (PA) remodeling is their common feature, in addition to bone morphogenetic protein receptor type 2; it is elusive whether there are other novel common genes and similar underlying mechanisms. To identify novel common hub genes involved in PA remodeling at different PH groups, we analyzed mRNA-Seq data located in the general gene expression profile GSE130391 utilizing bioinformatics technology. This database contains PA samples from different PH groups of hospitalized patients with chronic thromboembolic pulmonary hypertension (CTEPH), idiopathic pulmonary artery hypertension (IPAH), and PA samples from organ donors without known pulmonary vascular diseases as control. We screened 22 hub genes that affect PA remodeling, most of which have not been reported in PH. We verified the top 10 common hub genes in hypoxia with Sugen-induced PAH rat models by qRT-PCR. The three upregulated candidate genes are *WASF1, ARHGEF1* and *RB1* and the seven downregulated candidate genes are *IL1R1, RHOB, DAPK1, TNFAIP6, PKN1, PLOD2*, and *MYOF*. *WASF1, ARHGEF1*, and *RB1* were upregulated significantly in hypoxia with Sugen-induced PAH, while *IL1R1, DAPK1*, and *TNFA1P6* were upregulated significantly in hypoxia with Sugen-induced PAH. The DEGs detected by mRNA-Seq in hospitalized patients with PH are different from those in animal models. This study will provide some novel target genes to further study PH mechanisms and treatment.

## Introduction

Pulmonary hypertension (PH) is a multifaceted disease with a high mortality rate and a low cure rate, characterized by remodeling of the pulmonary artery (PA), which leads to elevated mean pulmonary artery pressure (mPAP), increased pulmonary vascular resistance (PVR), and eventually result in right heart failure (1, 2). According to WHO, PH can be divided into five categories based on pathological features, hemodynamic characteristics, clinical diagnosis, and treatment strategies (3, 4). Although the etiology of PH varies, PA remodeling is a common feature in different groups of PH. The injured pulmonary artery endothelial cells (PAECs) and the phenotype conversion of pulmonary artery smooth muscle cells (PASMCs), which are characterized by excessive proliferation and apoptosis resistance, play vital roles in PA remodeling. Also, the activation of fibroblasts causes the hypersecretion of extracellular matrices, such as collagen, resulting in extracellular matrix deposition, and subsequently, contributing to pulmonary vascular remodeling. Taken together, alterations in the aforementioned cells play three important roles in pulmonary vascular remodeling that contributes to the progression of PH (1–4).

One typical example of common genes is bone morphogenetic protein receptor type 2 (BMPR2), which was first revealed in patients with hereditary PAH and patients with IPAH (5–7), and then validated in the development of group 3 PH (8), chronic thromboembolic pulmonary hypertension (8, 9), valvular heart disease complicated with pulmonary artery hypertension (10), and hypoxia-induced animal model (11, 12). However, the other novel common genes and mechanisms of PA remodeling in different groups of PH are not fully elucidated; hence, it is vital to explore its pathogenesis further. With the development of gene chip technology, the database of gene expression profiles has grown dramatically. There have been some genetic bioinformatics studies utilizing the samples of patients with PH and animal models; most of them are focus studies on a single group of PH, such as idiopathic pulmonary artery hypertension (IPAH, Group 1) (13–16), hypoxia-induced pulmonary hypertension (Group 3) (17), and chronic thromboembolic pulmonary hypertension (CTEPH, Group 4) (18, 19). However, there are few reports on the comparative analysis among different groups of PH.

Some scientists used samples including blood (20, 21) or lung tissue samples (13–18, 22, 23) to indirectly explore genetic changes in PA remodeling. Recently, Gorr et al. identified DEGs between PASMCs derived from patients with IPAH and normal, and they were not sure whether it applies to other PH groups (24). Halliday et al. and Wang et al. directly used human PA as the research object and identified DEGs between CTEPH and the normal groups (18, 19). The common DEGs of PH in different groups have received less attention. Therefore, it is vital to further explore the common genes that are involved in the pathogenesis of PH.

In this study, GSE130391 chip data were selected as the research object, including the gene chip data of pulmonary arteries of organ donors without known pulmonary vascular diseases, patients with IPAH, and patients with CTEPH. First, we compared the differences and similarities between IPAH and CTEPH and screened their common DEGs. Furthermore, the screened common DEGs were verified using hypoxia with Sugen-induced PAH (Group 1).

## Materials and methods

### Bioinformatic analysis

The datasets of gene expression profiles with sequence numbers of GSE130391 were downloaded from the Gene Expression Omnibus (GEO) database, affiliated with the National Center for Biotechnology Information (NCBI) (25, 26), using the geoquery package; the details of the chips are listed in Table 1. The limma package in R software (version 3.6.3) (University of California, Berkeley, CA) was used to analyze the DEGs between the two groups (27, 28). The ComplexHeatmap package in R software was subsequently used to plot the heatmap of the DEGs (29).

**Table 1:** Detailed data of GSE130391.

**Sequence number of chip**	**GSE130391**
Platform	GPL570
Disease	PH (IPAH and CTEPH)
Chip provider	Pulmonary Lab of Medicine of Vanderbilt University
Address	4200 E 9TH Ave, Box B133, Nashville, TN, USA
Research object	Human
Sample type	Pulmonary artery
Number of chip samples	4 normal, 4 IPAH, 14 CTEPH
Time of uploading chip	Apr 29, 2019

Gene ontology enrichment (GO) and KEGG (Kyoto Encyclopedia of Genes and Genomes) pathway analyses of DEGs were performed *via* R software (version 3.6.3) (statistical analysis and visualization). The clusterProfiler package was primarily used for enrichment analysis (30); Org.hs.eg.db package (version 3.10.0) was used for ID conversion (31). GO analysis consists of biological processes (BP), cellular components (CC), and molecular functions (MF).

The PPI network of DEGs was constructed using STRING (https://string-db.org/, cut-off >0.4) (32). The significant modules (MCODE, degree cut-off = 2, node score cut-off = 0.2) and the hub genes were identified using Cytoscape software (33, 34). The gene expression level and protein expression scores were from the Human Protein Atlas (HPA) database and the Bgee database.

### Hypoxia with Sugen-induced PH model

Adult male Sprague Dawley rats (180–200 g, 8 weeks old) were supplied by the Laboratory Animal Center of Tongji Medical College, Huazhong University of Science and Technology (HUST). The animal study protocol was reviewed and permitted by HUST Animal Care and Use Committee and conducted following the Guidelines for the Care and Use of Laboratory Animals (S1821). They were injected with 20 mg/kg Sugen 5416 (HY-10374, MCE) subcutaneously and exposed to 10% oxygen for 2 weeks; control rats were injected with vehicle (0.5% carboxymethylcellulose, 0.9% NaCl, 0.4% polysorbate, and 0.9% benzyl alcohol in deionized water) (35). As we recently described, hemodynamic studies were performed (35–39). At the end of hemodynamic experiments, the rats were euthanized with anesthetics, the lungs and heart tissues were collected, and a part of the lung was soaked in a formaldehyde solution (4%) for 24 h. The right ventricular hypertrophy was quantified by weighing the right ventricular free wall (RV) and the left ventricle (LV) together with the septum (S, LV+S) (35–39). The rest of the lungs were stored at −80°C for further analysis. Five-micrometer-thick sections of the lung embedded with paraffin were used to assess PA remodeling after HE-stained. As described in our previous studies, the wall thickness in PAs was determined to be around 50–100 μm diameters (35–39).

### Real-time quantitative RT-PCR

The pulmonary arteries were isolated from the third branch of the intralobular artery in the lung of rat models as described in our previous publications (35–39). RNA extraction (ratio of OD at 260 nm/280 nm > 1.7) was conducted using the Trizol method (15596026, Invitrogen) and reversely transcribed into cDNA according to the manufacturer's recommendations (RR037A, Takara). Candidate primers for each gene of interest were designed using the Premier 5 design program (VertMarkets, Inc., Pennsylvania). The best candidate primers were then selected using BLAST alignment. PCR reaction was performed with the quantitative TB Green-based PCR kit (RR420A, Takara) using a CFX Connect PCR machine (CFX Connect TM, BIO-RAD). The following conditions were set for the reaction: pre-denaturation stage: 95°C, 1 min/1 cycle; amplification stage: denaturation at 95°C for 5 s and annealing at 58°C for 30 s, 40 cycles; melting curve stage: 65–95°C, increment of 0.5°C for 5 s. Results were analyzed using the 2^−ΔΔCT^ method (40, 41). The primer sequences for each gene are listed in Table 2.

**Table 2:** The q-PCR primer of top 10 hub common DEGs.

**Target gene**	**q-PCR primer**
1L1R1 (rat)	F: 5′-CTGCCGAGGCTTGTGACATCTTC-3′
	R: 5′-CGACAGCAGAGGCACAATGAGAC-3′
RHOB (rat)	F: 5′-CATCCGCAAGAAGCTGGTGGTG-3′
	R: 5′-TCGGGGAACTCGTCCTTACTGAAC-3′
DAPK1 (rat)	F: 5′-CGACAGCAGAGGCACAATGAGAC-3′
	R: 5′-GCTCCTCACGCTCACATTCTCAC-3′
TNFAIP6 (rat)	F: 5′-GACGATGTCCACGGCTTTGTAGG-3′
	R: 5′-ACGGACGCATCACTCAGAAACTTC-3′
PKN1 (rat)	F: 5′-ACCTTCCCGAGACCATCCCTTG-3′
	R: 5′-TGAGGCTGCTCCGTCCACTAAG-3′
PLOD2 (rat)	F: 5′-CGTCTGGCTTCACTTTATCCGAGAG-3′
	R: 5′-TACGAAGTTCAGCAGGGCAAATCC-3′
MYOF (rat)	F: 5′-GCCTAGAGAAGCAGAAGCACAGTG-3′
	R: 5′-AGCAGCGTAGGTGGTAGATGTAGAC-3′
WASF1 (rat)	F: 5′-ACAAGTCCTGCCTGCCTCTGAG-3′
	R: 5′-CTTCCTGTTCACGCTGCTCTTCC-3′
ARHGEF1 (rat)	F: 5′-CCTGAGCCTGGAGATGATGGAGAG-3′
	R: 5′-TGCCGCTTCACTTGGTTCTTGG-3′
RB1 (rat)	F: 5′-CCTCCCTCTCCAGAGTAACCACAC-3′
	R: 5′-GTATGGAAGGCTGAGGCTGCTTG-3′

### Western blot

Total proteins were extracted from PAs in control rat models using RIPA lysis buffer containing PMSF and phosphatase inhibitors. Proteins (30 μg/well) were electrophoresed using a 10% sodium dodecyl sulfate (SDS) polyacrylamide gel, as described in our previous studies (39, 42), and transferred to PVDF membranes (Millipore, Germany). The membranes were blocked with 3% BSA for 2 h at room temperature and then incubated with anti-RB1 antibody (1:500, R22869, ZEN BIO, China), anti-ARHGEF1 antibody (1:500, PB10045, BOSTER, China), anti-WASF1 antibody (1:500, R26102, ZEN BIO, China), and anti-β-actin (1:10,000, A01010, Abbkine, China) overnight. Bands were detected with ECL Substrate (34580, Thermo Scientific). Integrated density analysis was performed with ImageJ software.

### Statistical analysis

Data are the mean ± SE and t-test (and non-parametric tests) was used for two-group comparisons. *p* < 0.05 represents significant differences.

## Results

### Identification and comparison of DEGs between IPAH and CTEPH

**Figure 1:**
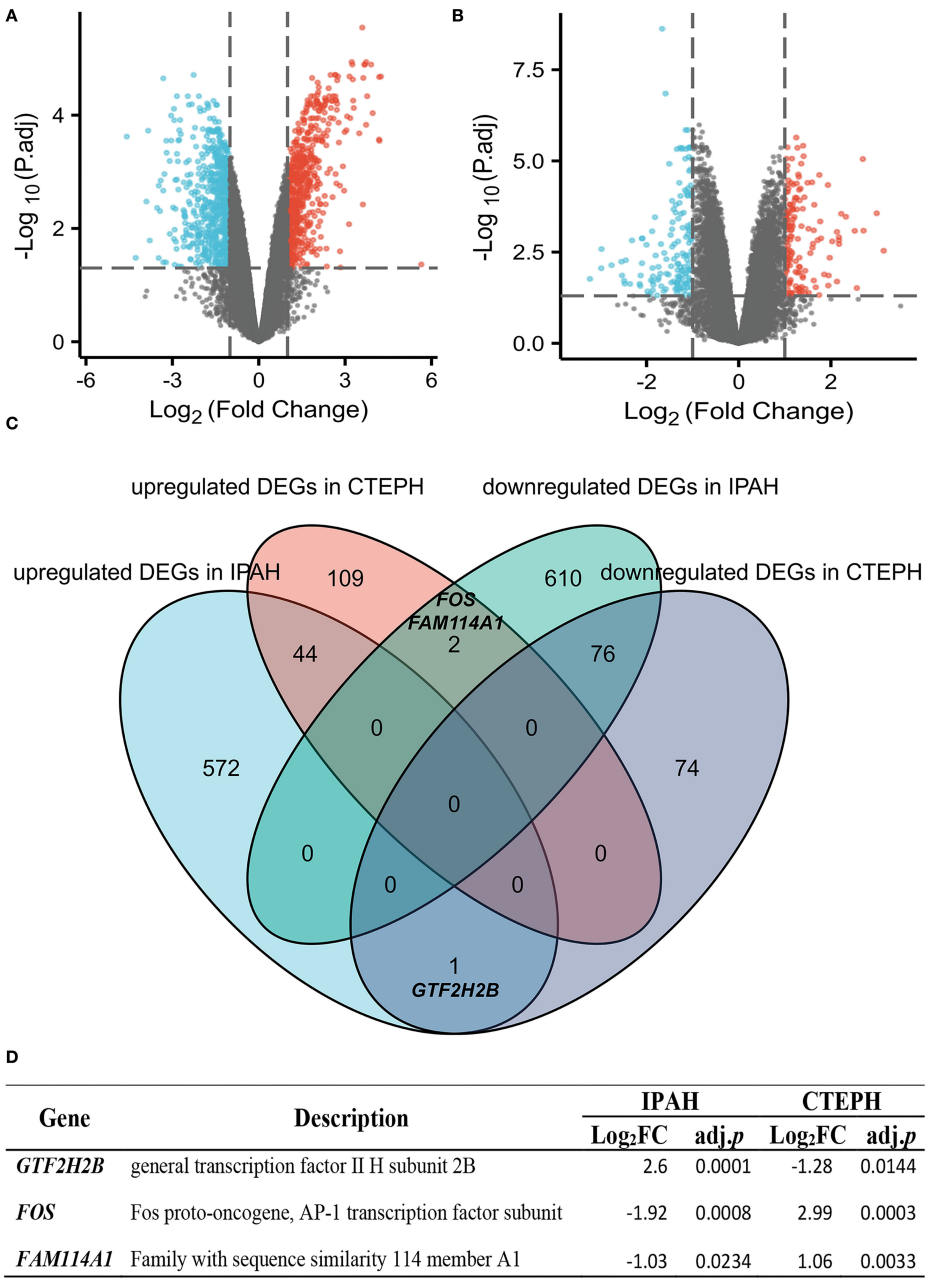
Identification and comparative analysis of DEGs in IPAH and CTEPH. **(A,B)** Volcano plot analysis identifying DEGs. Red denotes upregulated gene and blue signifies downregulated gene from PA samples from patients with IPAH and CTEPH (|logFC| > 1, adj. *p* < 0.05). **(C)** Downregulated and upregulated DEGs overlap between IPAH and CTEPH by the bioinformatic analysis were illustrated in the Venn diagram. **(D)** The detail of three special overlapping DEGs between IPAH and CTEPH.

A total of 1,305 differential genes were screened between normal and IPAH subjects, including 617 genes with upregulated expression and 688 with downregulated expression, which was statistically significant (|logFC| > 1, adj. *p* < 0.05), as illustrated in the volcano map (Figure 1A). A total of 306 differential genes were screened between normal and CTEPH, including 155 genes with upregulated expression and 151 with downregulated expression, which was statistically significant (|logFC| > 1, adj. *p* < 0.05), as depicted in the volcano map (Figure 1B). Seventy-six overlapping genes with downregulated expression and 44 overlapping genes with upregulated expression were common for IPAH and CTEPH (Figure 1C). However, if the expressions are not distinguished, there are 123 common DEGs in total as shown in Figure 1C. This result shows that three differential genes (*GTF2H2B, FOS*, and *FAM114A1*) are upregulated in one group and downregulated in the other group (Figures 1C,D). The general transcription factor II H subunit 2B (*GTF2H2B*) was upregulated in IPAH (Log_2_FC = 2.60, adj. *p* = 0.0001), but downregulated in CTEPH (Log_2_FC = −1.28, adj. *p* = 0.0144). Fos proto-oncogene, AP-1 transcription factor subunit (*FOS*) was downregulated in IPAH (Log_2_FC = −1.92, adj. *p* = 0.0008), but upregulated in CTEPH (Log_2_FC = 2.99, adj. *p* = 0.0003) as Wang et al. reported (19). Family with sequence similarity 114 member A1 (*FAM114A1*) was downregulated in IPAH (Log_2_FC = −1.03, adj. *p* = 0.0234), but upregulated in CTEPH (Log_2_FC = 1.06, adj. *p* = 0.0033).

### Biological features between IPAH and CTEPH

**Figure 2:**
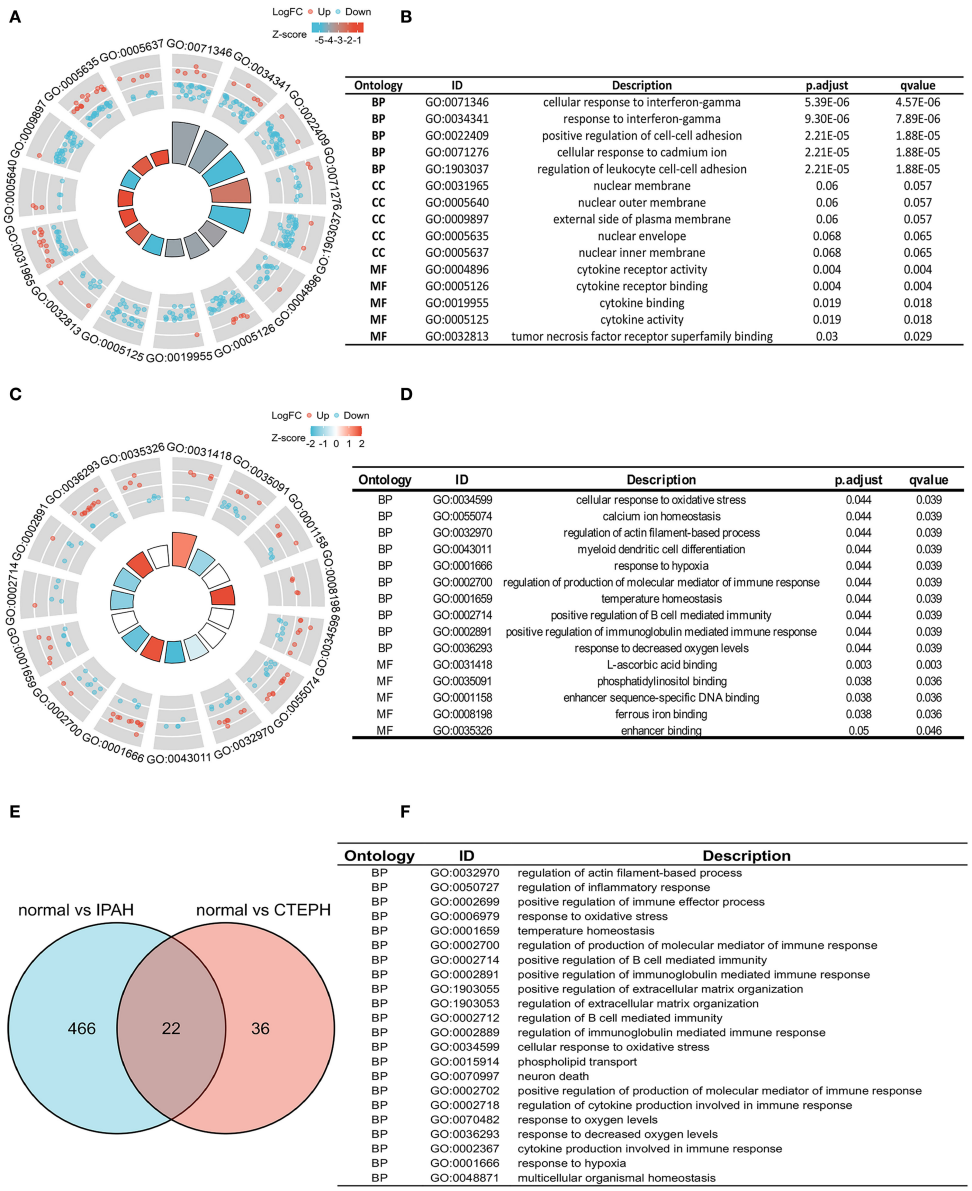
Biological features in IPAH and CTEPH. **(A,B)** A part of the GO enrichment results shows the DEGs in IPAH (adj. *p* < 0.05 and *q* < 0.2). **(C,D)** A part of the GO enrichment results showing the DEGs in CTEPH (adj. *p* < 0.05 and *q* < 0.2). **(E,F)** The overlapping GO of DEGs between IPAH and CTEPH in the Venn diagram and the detail of 22 overlapping GOs of DEGs between IPAH and CTEPH. BP, biological processes; CC, cellular components, and MF, molecular functions.

To investigate the biological features of the DEGs, we applied the GO analysis. There were 488 results relative to IPAH, which included 474 BPs, 8 CCs, and 6 MFs. The top five of each ontology that changed are shown in Figure 2A. Regarding BPs of the DEGs of IPAH, these included: significant enrichment in the cellular response to interferon-gamma, response to interferon-gamma, positive regulation of cell–cell adhesion, cellular response to cadmium ion, and regulation of leukocyte cell–cell adhesion (Figures 2A,B; adj. *p* < 0.05 and *q* < 0.2). Regarding CCs of the DEGs of IPAH, these included: significant enrichment in the nuclear membrane, nuclear outer membrane, external side of the plasma membrane, nuclear envelope, and nuclear inner membrane (Figures 2A,B; adj. *p* < 0.05 and *q* < 0.2). Regarding MFs of the DEGs of IPAH, these included: significant enrichment in cytokine receptor activity, cytokine receptor binding, cytokine binding, cytokine activity, and tumor necrosis factor receptor superfamily binding (Figures 2A,B; adj. *p* < 0.05 and *q* < 0.2). These results mainly indicated that some DEGs' products on the nuclear membrane are involved in the cellular response to interferon-gamma in IPAH through binding cytokine-related proteins.

GO analysis identified 58 results in CTEPH, including 50 BPs and 8 MFs. The changes in BPs of DEGs included: enrichment in cellular responses to oxidative stress, calcium ion homeostasis, regulation of actin filament-based processes, myeloid dendritic cell differentiation, and responses to hypoxia (Figures 2C,D; adj. *p* < 0.05 and *q* < 0.2). The changes in MF of the DEGs included: significant enrichment in L-ascorbic acid binding, phosphatidylinositol binding, enhancer sequence-specific DNA binding, ferrous iron binding, and enhancer binding, as shown in Figures 2C,D. These results suggested that some of the DEG products are involved in oxidative stress, calcium ion homeostasis, and responses to hypoxia in CTEPH through binding phosphatidylinositol proteins.

We also found 22 overlapping functional annotations in IPAH and CTEPH (Figure 2E). They mainly included regulation of actin filament-based process, regulation of inflammatory response, response to oxidative stress, and positive regulation of extracellular matrix organization (Figure 2F). There have been numerous studies related to these biological processes involved in IPAH and CTEPH.

### Possible mechanisms between IPAH and CTEPH

**Figure 3:**
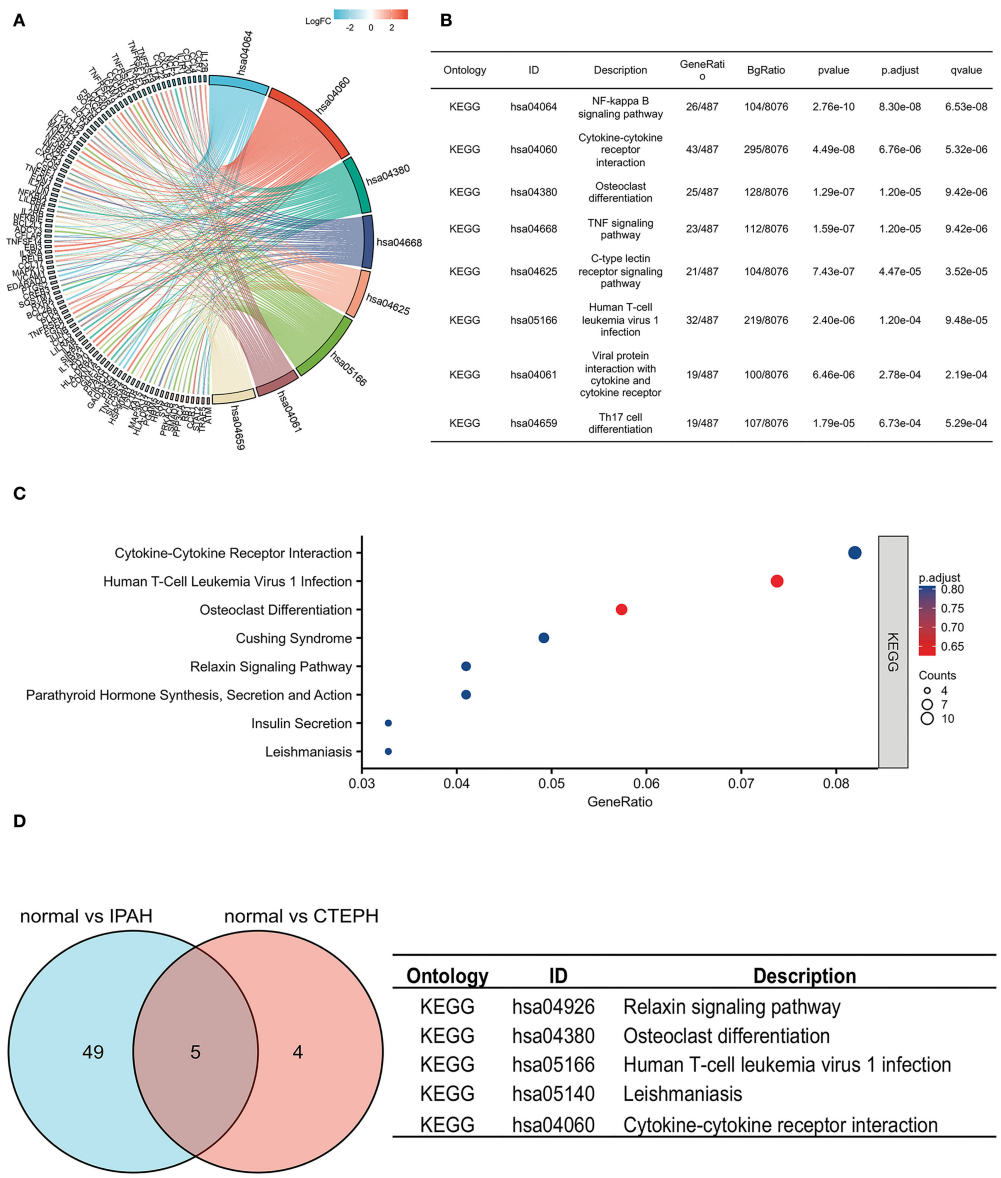
KEGG pathways in IPAH and CTEPH. **(A,B)** KEGG pathways analysis of DEGs in IPAH (adj. *p* < 0.05 and *q* < 0.2). **(C)** KEGG pathways analysis of DEGs in CTEPH (gene count >2 and *p* < 0.05). **(D)** The overlapping KEGG pathways of DEGs between IPAH and CTEPH in the Venn diagram and the detail of five overlapping KEGG pathways of DEGs between IPAH and CTEPH.

To investigate the possible mechanisms of these DEGs, a KEGG pathway analysis indicated that the DEGs of IPAH were involved in the NF-κB signaling pathway, cytokine–cytokine receptor interaction, osteoclast differentiation, the TNF (tumor necrosis factor) signaling pathway, the C-type lectin receptor signaling pathway, human T-cell leukemia virus 1 infection, viral protein interaction with cytokine and cytokine receptor, and Th17 cell differentiation, under the conditions of adj. *p* < 0.05 and *q* < 0.2 (Figures 3A,B). There was no pathway identified under the conditions of adj. *p* < 0.05 and *q* < 0.2 in DEGs of CTEPH. However, when the threshold was gene count >2 and *p* < 0.05, the pathway was majorly enriched in cytokine–cytokine receptor interactions; osteoclast differentiation; human T-cell leukemia virus 1 infection; Cushing syndrome; the relaxin signaling pathway; parathyroid hormone synthesis, secretion, and action; insulin secretion; and leishmaniasis (Figure 3C). There are five overlapping pathways (osteoclast differentiation, human T-cell leukemia virus 1 infection, cytokine-cytokine receptor interaction, leishmaniasis, and relaxin signaling pathway) in IPAH and CTEPH. GO and KEGG analysis showed that their PA remodelings share some mechanisms.

### The top 10 hub genes between IPAH and CTEPH

**Figure 4:**
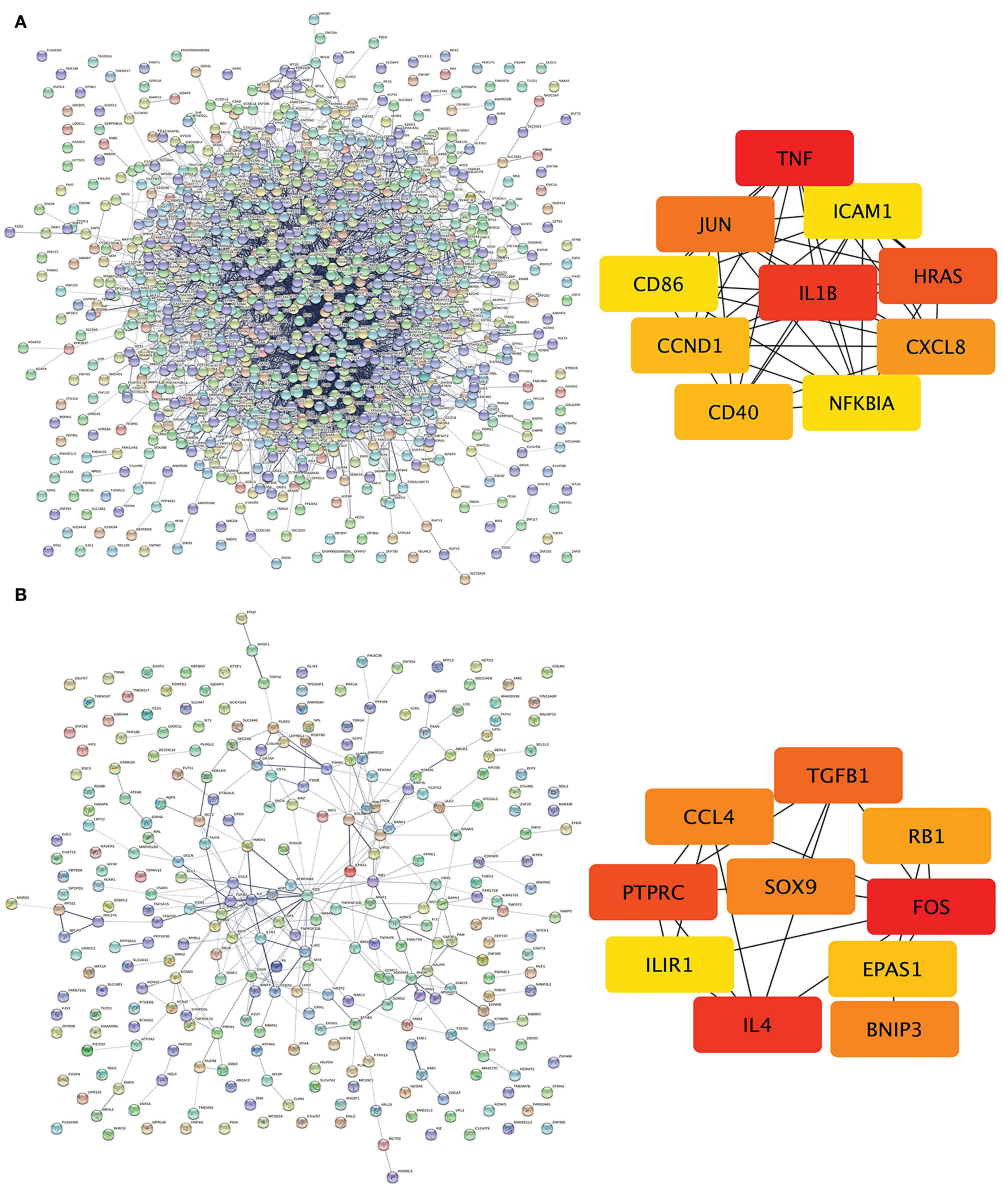
PPI networks and modules in IPAH and CTEPH. **(A)** STRING software was used to analyze the PPI network of 1,305 DEGs in IPAH (*left*). There were 1,080 nodes and 4,940 edges. Cytohubba identified the top 10 hub genes in IPAH (*right*). **(B)** STRING software was used to analyze the PPI network of 306 DEGs in CTEPH (*left*). There were 274 nodes and 285 edges. Cytohubba identified the top 10 hub genes in CTEPH (*right*).

The PPI network of DEGs in IPAH is shown on the left of Figure 4A. The top 10 hub genes include *TNF, IL1B, HRAS, JUN, CCXL8, CD40, CCND1, ICAM1, NFKBIA*, and *CD86* (Figure 4A, *right*; Table 3). The PPI network of DEGs in CTEPH is shown on the left of Figure 4B. The top 10 hub genes included five downregulated genes (*IL4, TGFB1, SOX9, CCL4*, and *EPAS1*) and five upregulated genes (*FOS, PTPRC, BNIP3, RB1*, and *IL1R1)* (Figure 4A, *right*; Table 4).

**Table 3:** Top 10 hub genes in IPAH.

**Gene**	**Description**	**Degree of connectivity**	**IPAH (logFC)**
TNF	Tumor necrosis factor	146	−1.76752525
IL1B	Interleukin 1 beta	116	−2.25446375
HRAS	HRas proto-oncogene, GTPase	99	−1.000599
JUN	Jun proto-oncogene, AP-1 transcription factor subunit	94	−1.85835925
CXCL8	C-X-C motif chemokine ligand 8	84	−3.19954825
CD40	CD40 molecule	75	−2.28042175
CCND1	cyclin D1	75	−2.58089625
ICAM1	Intercellular adhesion molecule 1	74	−3.47023425
NFKBIA	NFKB inhibitor alpha	74	−1.80282
CD86	CD86 molecule	74	−1.11225075

**Table 4:** Top 10 hub genes in CTEPH.

**Gene**	**Description**	**Degree of connectivity**	**CTEPH (logFC)**
FOS	Fos proto-oncogene, AP-1 transcription factor subunit	24	2.994168071
IL4	Interleukin 4	18	−1.0257366
PTPRC	Protein tyrosine phosphatase receptor type C	17	1.226277071
TGFB1	Transforming growth factor beta 1	12	−1.587007571
SOX9	SRY-box transcription factor 9	11	−1.585361429
CCL4	C-C motif chemokine ligand 4	11	−1.301053857
BNIP3	BCL2 interacting protein 3	11	1.159415
RB1	RB transcriptional corepressor 1	10	1.05062846
EPAS1	Endothelial PAS domain protein 1	9	−1.459483393
IL1R1	Interleukin 1 receptor type 1	8	2.485151893

### Screening the common DEGs in IPAH and CTEPH

**Figure 5:**
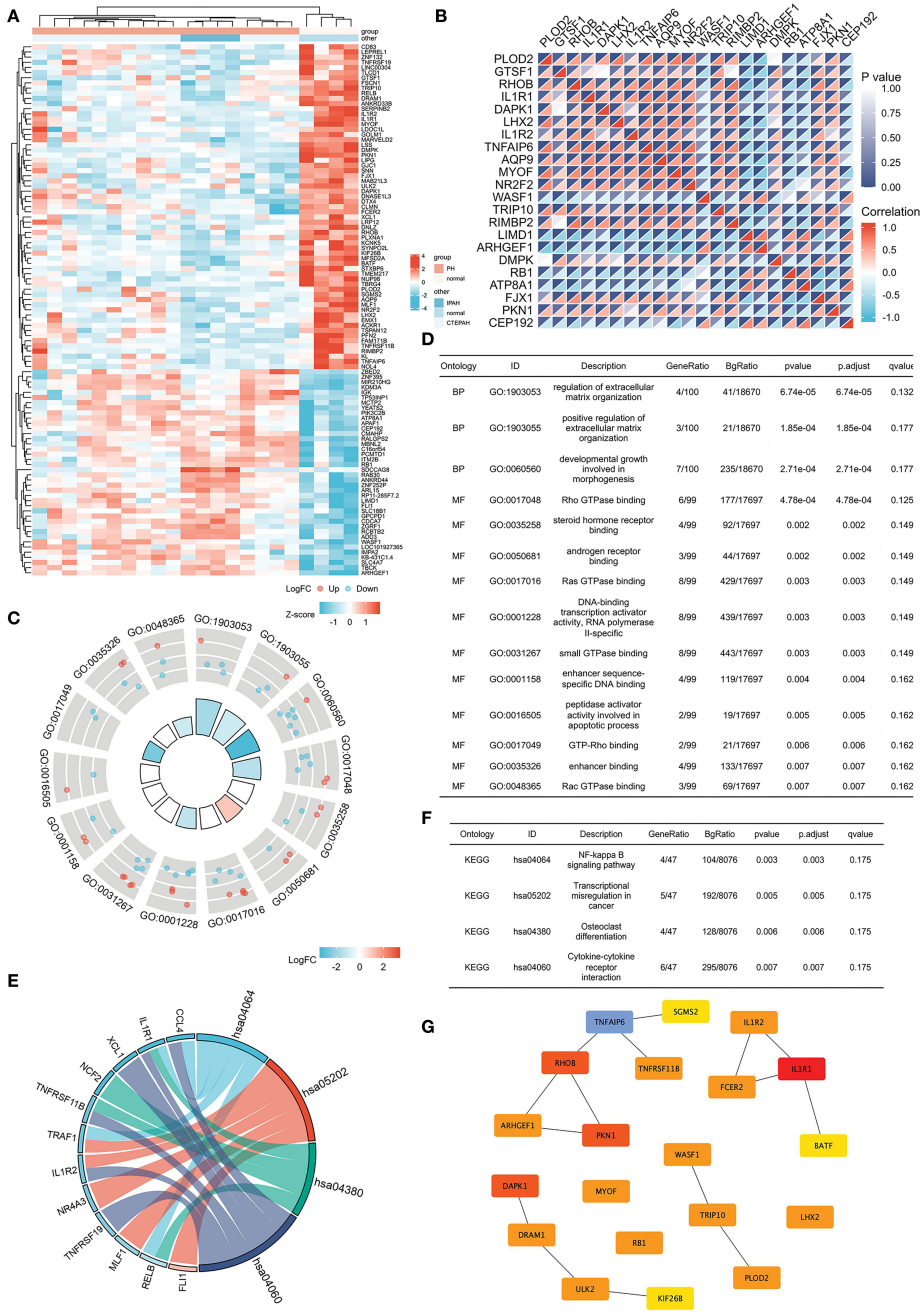
Common DEGs in IPAH and CTEPH and the hub genes of common DEGs. **(A)** Heatmap of the common 120 DEGs in IPAH and CTEPH. Red areas denote increased gene expressions, and blue areas denote decreased gene expressions in PA compared to normal. **(B)** In the correlation heatmap, abscissa and ordinate indicate 22 DEGs features by Spearman correlation analysis. The absolute value of the correlation coefficient is displayed on the right side of the color scale (from −1 to 1, and from blue to red). **(C,D)** GO enrichment result of the common DEGs. **(E,F)** KEGG pathway analysis of the common DEGs. **(G)** The most significant modules are identified in IPAH and CTEPH by Cytoscape.

The Venn diagram shows overlapping genes among normal vs. IPAH and normal vs. CTEPH in Figure 1A. Venn diagram showed that 76 common downregulated DEGs and 44 common upregulated DEGs overlapped in IPAH and CTEPH (|logFC| > 1, adjusted *p* < 0.05), which means that these genes do not differ between them, shown in the heatmap (Figure 5A). Furthermore, the correlation heat map analyzed the important patterns and relationships among common DEGs by Spearman (Figure 5B).

GO analysis revealed that the common DEGs were chiefly enhanced in the regulation of extracellular matrix organization, Ras GTPase binding, Rho GTPase binding, small GTPase binding, and androgen receptor binding (adj. *p* < 0.05 and *q* < 0.2), as shown in Figures 5C,D. KEGG pathway analysis showed that the pathway was mainly enhanced in NF-κB signaling pathway, transcriptional misregulation in cancer, osteoclast differentiation, and cytokine–cytokine receptor interactions (Figures 5E,F). Some of these functional features and pathways have been extensively researched in pulmonary hypertension, which corroborated our findings.

The most significant modules are shown in Figure 5G. Based on the Venn diagram, gene expression levels, and scores, we identified and screened the top 22 hub genes by Cytoscape, including seven upregulated genes, *WASF1, ARHGEF1, RB1, ITM2B, ADD3, APAF1*, and *LIMD1*, and 15 downregulated genes, *IL1R1, RHOB, DAPK1, TNFAIP6, PKN1, PLOD2, TRIP10, MYOF, DRAM1, ULK2, FSCN1, NR2F2, PFN2, DMPK*, and *LPR12* (Table 5).

**Table 5:** Top 22 hub genes of common DEGs in IPAH and CTEPH.

**Gene**	**Description**	**Degree of connectivity**	**IPAH**	**CTEPH**
			**(logFC)**	**(logFC)**
IL1R1	Interleukin 1 receptor type 1	4	−3.31181275	−2.485151893
RHOB	Ras homolog family member B	3	−3.429416	−2.315058
DAPK1	Death-associated protein kinase 1	3	−3.03807425	−1.965708857
TNFAIP6	TNF alpha-induced protein 6	3	−2.6127775	−1.741152464
PKN1	Protein kinase N1	3	−1.0623545	−1.105080357
PLOD2	Procollagen-lysine,2-oxoglutarate 5-dioxygenase 2	2	−3.901955	−2.530431857
MYOF	Myoferlin	2	−2.12081	−1.569080464
WASF1	WASP family member 1	2	1.783399	2.03028325
ARHGEF1	Rho guanine nucleotide exchange factor 1	2	1.35552975	1.034979714
RB1	RB transcriptional corepressor 1	2	1.31365525	1.050628464
TRIP10	Thyroid hormone receptor interactor 10	2	−1.630505	−1.284504071
DRAM1	DNA damage regulated autophagy modulator 1	2	−1.5938555	−1.022801571
ULK2	Unc-51 like autophagy activating kinase 2	2	−1.11539675	−1.816644464
ITM2B	Integral membrane protein 2B	1	1.235915286	1.727951
ADD3	Adducin 3	1	2.0188955	1.039967571
APAF1	Apoptotic peptidase activating factor 1	1	1.19376925	1.264531857
LIMD1	LIM domain containing 1	1	1.170496964	1.46983275
FSCN1	Fascin actin-bundling protein 1	1	−2.10963625	−1.261688929
NR2F2	Nuclear receptor subfamily 2 group F member 2	1	−1.8798225	−1.151422321
PFN2	Profilin 2	1	−2.1160365	−1.615431393
DMPK	Dystrophia myotonica-protein kinase	1	−1.35368875	−1.661622036
LRP12	Low density lipoprotein receptor-related protein 12	1	−2.789589	−2.543066786

**Figure 6:**
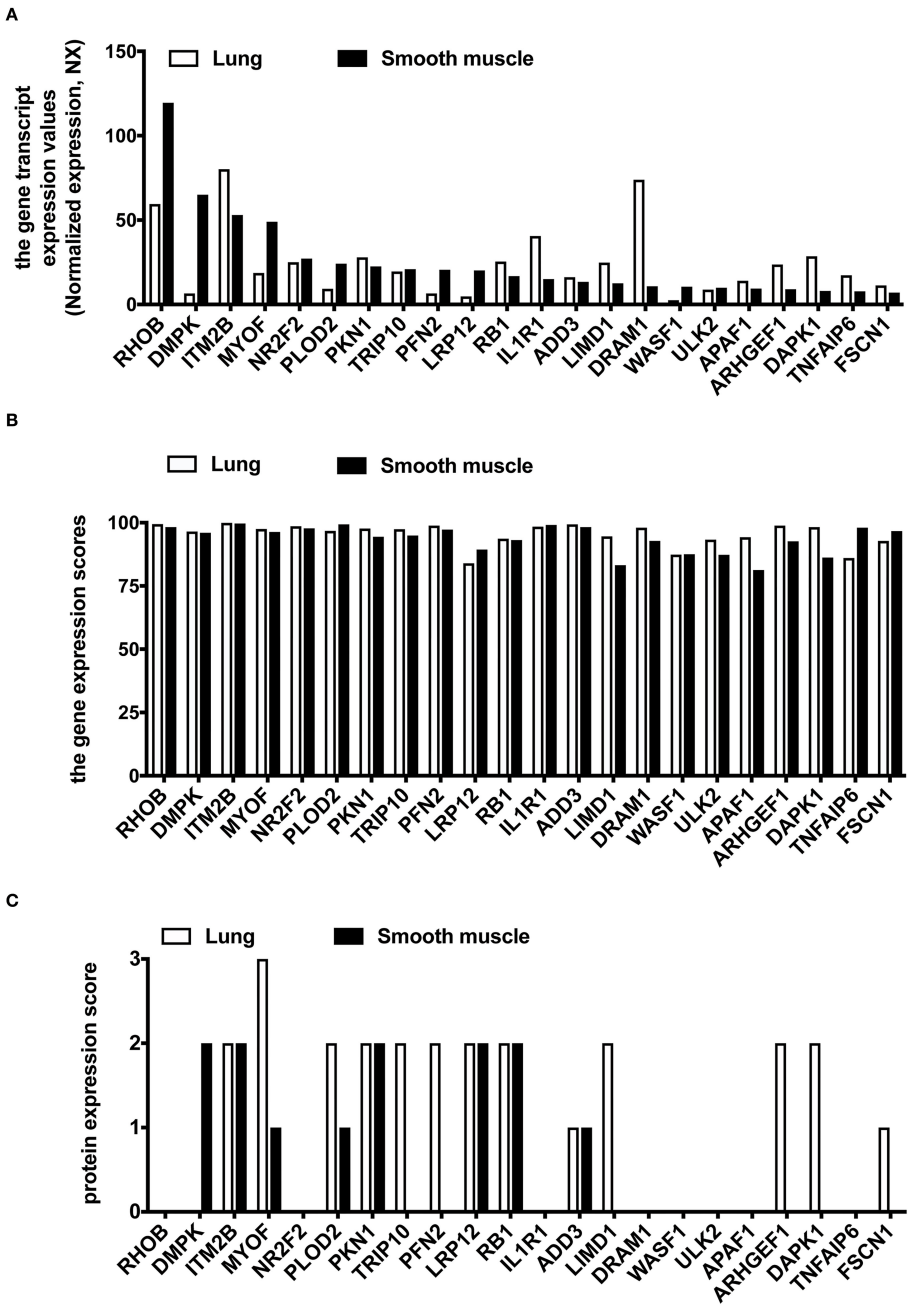
The expression analysis of 22 common hub DEGs in human lung and smooth muscle tissues. **(A)** Gene expression level of hub genes in HPA database. **(B)** Gene expression scores of hub genes were compared in the Bgee database. **(C)** Protein expression of hub genes in HPA database.

We used the HPA database to compare the gene expression levels of these 22 hub genes between the human lung and smooth muscle tissue. This served as a primary reference for determining whether these 22 hub genes were expressed in lung and smooth muscle tissue. The findings revealed that the gene expression of *RHOB, DMPK, MYOF, NR2F2, PLOD2, TRIP10, PFN2, LRP12, WASF1*, and *ULK2* were lower in the lung compared to the smooth muscle (Figure 6A). We then analyzed the gene expression scores of these 22 hub genes in the lung and the smooth muscle tissue from the Bgee database. All gene expression scores in the lung and the smooth muscle tissue were high (Figure 6B). Among them are many proteins that have not been detected in the lung tissue or the smooth muscle tissue from the HPA database (Figure 6C).

### qRT-PCR verified the top 10 candidate common hub genes

**Figure 7:**
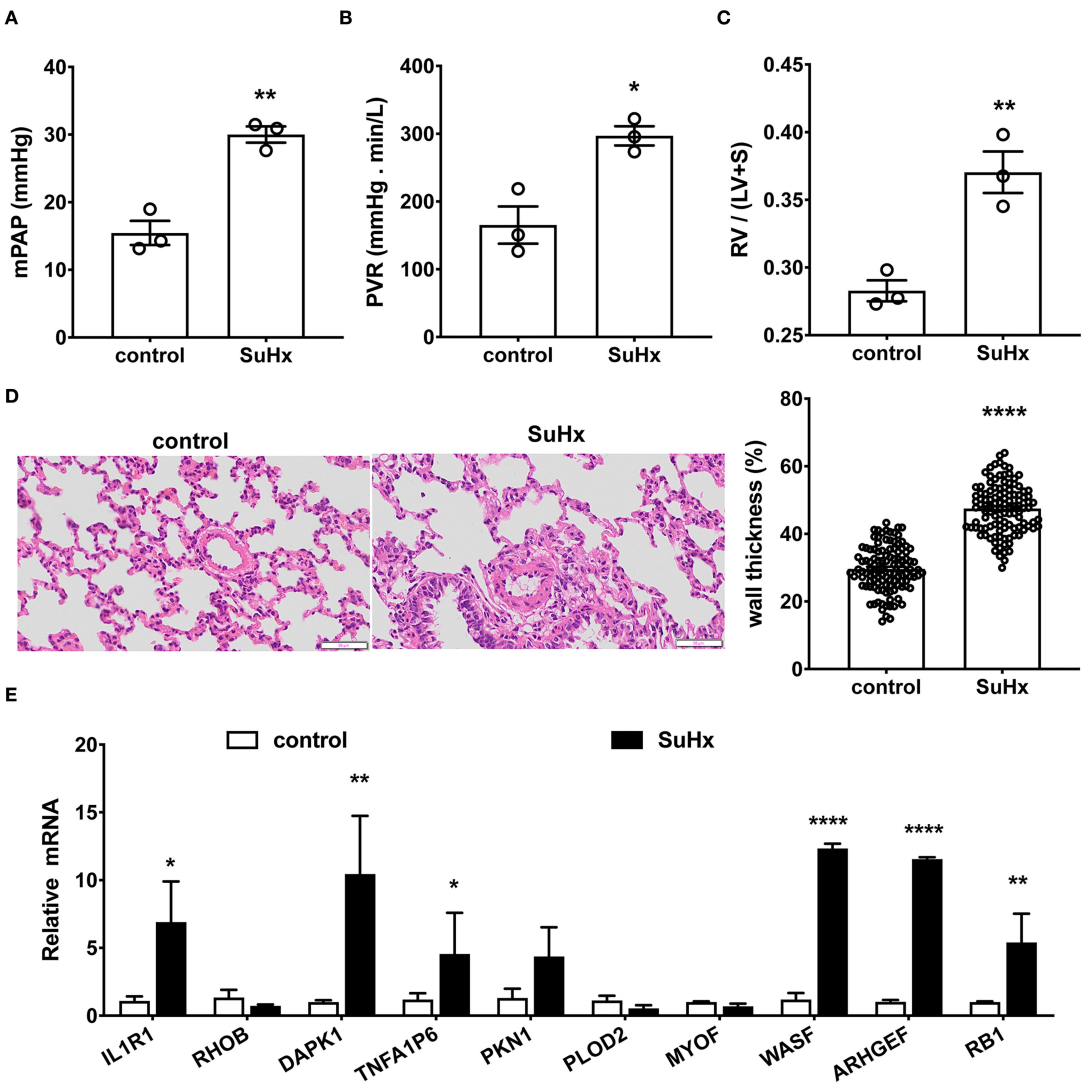
Verification of the top 10 hub genes in hypoxia with Sugen-induced PAH rats by qRT-PCR. **(A–D)** Hypoxia with Sugen-induced PAH (SuHx) characterized by increased mean pulmonary artery pressure [mPAP, **(A)**], pulmonary vascular resistance [PVR, **(B)**], hypertrophy of right ventricle [RV/(LV+S), **(C)**] and pulmonary artery [wall thickness, **(D)**]. Data are the mean ± SE, and t-test (and non-parametric tests) was used for two Comparisons. *n* = 3, * *p* < 0.05, ** *p* < 0.01, **** *p* < 0.0001, as compared with control (control rats). **(E)** The gene expression of the top 10 hub genes in the pulmonary artery was verified by qRT-PCR. *N* = 3, * *p* < 0.05; ** *p* < 0.01; **** *p* < 0.0001.

To verify if these top 10 hub genes do contribute to PA remodeling in any PH, we performed qRT-PCR to detect three upregulated candidate genes (*WASF1, ARHGEF1*, and *RB1*) and seven downregulated candidate genes (*IL1R1, RHOB, DAPK1, TNFAIP6, PKN1, PLOD2*, and *MYOF*), using hypoxia with Sugen-induced PAH classified into group 1 (Figure 7).

As shown in Figures 7A–D, in hypoxia with Sugen-induced PH rats (SuHx), mPAP (30.00 ± 0.97 mmHg for SuHx vs. 15.46 ± 1.46 mmHg for control rats; Figure 7A) and PVR (297.06 ± 11.50 mmHg min/L for SuHx, vs. 165.40 ± 22.50 mmHg min/L for control rats; Figure 7B) were remarkably elevated. RV/(LV+S) representing right heart hypertrophy increased significantly (0.37 ± 0.01 for SuHx vs. 0.28 ± 0.01 for control rats; Figure 7C). Pulmonary artery wall thickness representing PA remodeling was significantly increased (47.52 ± 0.67% for SuHx vs. 29.64 ± 0.62% for control rats, Figure 7D). qRT-PCR results showed six genes in these 10 common DEGs, which changed significantly after onset (Figure 7E). *WASF1, ARHGEF1*, and *RB1* were significantly upregulated, which was consistent with bioinformatics analysis, but *IL1R1, DAPK1*, and *TNFA1P6* were significantly upregulated, which was contrary to bioinformatics analysis.

### The protein expression of WASF1, ARHGEF1, and RB1

**Figure 8:**
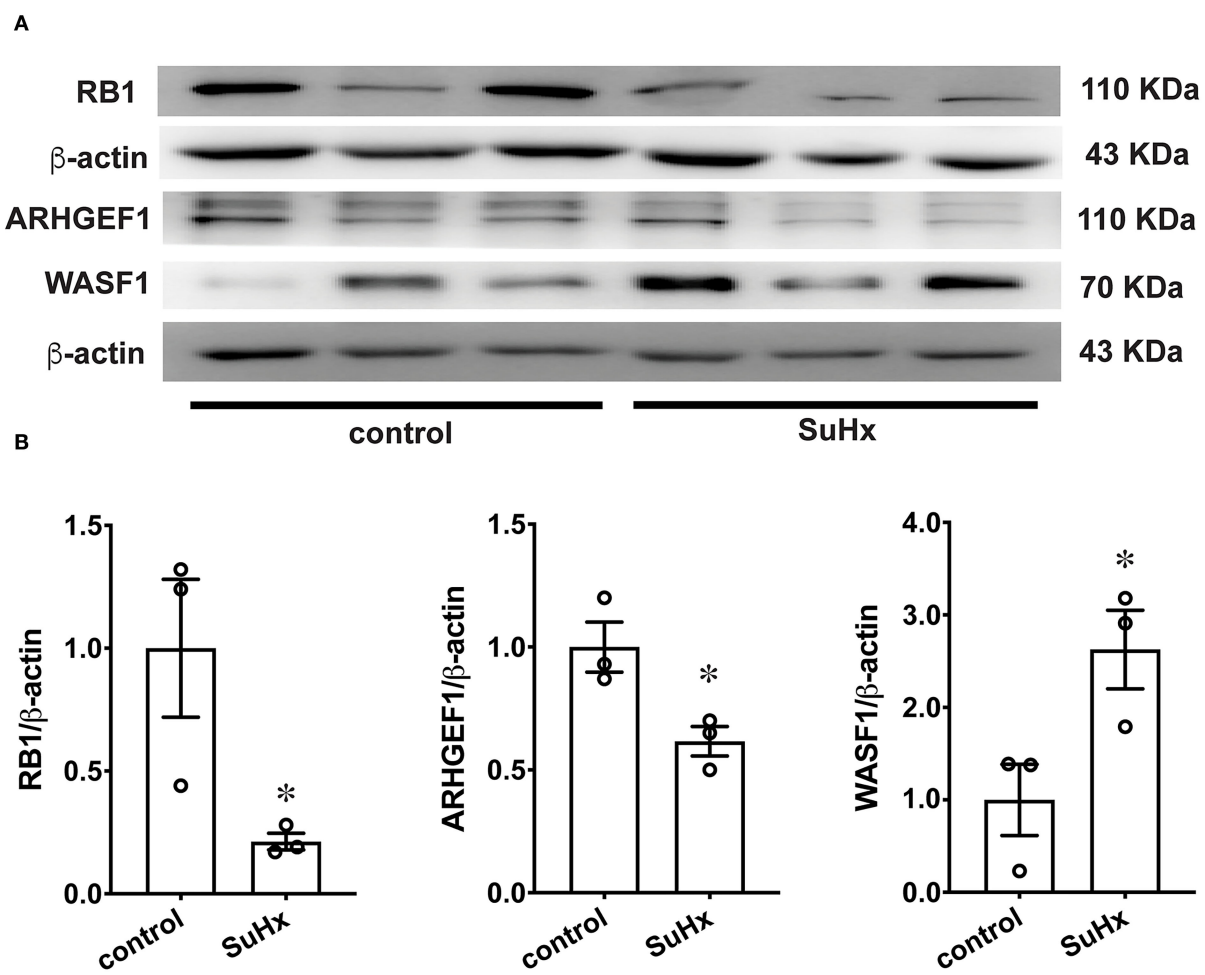
The protein expression of RB1, ARHGEF1, and WASF1 in model rat PA tissues. **(A)** Representative Western blot bands of RB1, ARHGEF1, and WASF1 in control and SuHx. **(B)** Integrated density analysis of the protein levels of RB1, ARHGEF1, and WASF1 in each group. *n* = 3, * *p* < 0.05.

To further verify the protein expression of WASF1, ARHGEF1, and RB1, we performed a western blot using rat PAs. The protein expression levels of WASF1 were significantly increased in the hypoxic environment with Sugen-induced PH rats, whereas ARHGEF1 and RB1 were significantly decreased in the same hypoxic environment (Figures 8A,B).

## Discussion

Pulmonary artery remodeling is one of the major processes for PH disease, although some characteristics of pulmonary artery remodeling vary in different PH groups. To better understand the mechanism of PA remodeling in the pathogenesis of PH disease, we analyzed the overlapping (common) DEGs associated with PA remodeling from a database containing samples of two PH group patients. By comparing the gene expression profiles between IPAH (Group 1) and CTEPH (Group 4) from the database, we identified overlapping sets of DEGs associated with vascular remodeling and enriched signal pathways between them. Among these, there are 76 overlapping genes in downregulated DEGs and 44 in upregulated DEGs between IPAH and CTEPH. Although *GTF2H2B, FOS*, and *FAM114A1* are also overlapping genes, they belong to upregulated gene clusters in one group and downregulated gene clusters in other groups. Increased FOS expression in primary PASMCs from patients with CTEPH was reported to be associated with excessive proliferation and migration of PASMCs (19). Consistently, FOS inhibitors attenuated TNF-α induced proliferation and migration in human PASMCs (19).

Meanwhile, to find out the common mechanisms and hub genes involved in PA remodeling in PH, we screened 22 hub DEGs and verified the top 10 common DEGs by using hypoxia with Sugen-induced PAH rats.

Among the 22 hub genes, it has been reported that *IL1R1* (43–46), *RHOB* (47, 48), *TNFAIP6* (49), *PFN2* (50), and *ARHGEF1* (51, 52) are involved in PA remodeling in different types of PH.

Previous studies have found that inflammation contributed to the development of PH, characterized by a significant increase in serum levels of interleukin (IL)-1 (43). Interleukin (IL)-1 participates in PA remodeling and inflammation during PH by binding to the receptor (IL1R1) which is upregulated and recruiting myeloid differentiation primary response protein 88 (MyD88), which induced IL-1, IL-6, and TNF-α increased through NF-κB activation in patients with IPAH and hypoxia-induced mice (44). IL-1R antagonist protects against murine PH secondary to bronchopulmonary dysplasia (BPD-PH) (45). But IL-1 stimulation reduced collagen expression in PASMCs and parenchymal fibroblasts and subsequently attenuated PA and interstitial remodeling in systemic sclerosis with concomitant PH (46). Therefore, the role of IL-1 is very complex. IL-1 promotes the proliferation of PASMC and parenchymal fibroblasts. However, IL-1 inhibits collagen, and α-SMA expressions and has direct antifibrotic activity. RhoB (Ras homologous gene family member β) acts synergistically with RhoA (Ras homologous gene family member α) on hypoxia-induced HIF-1α stability, vascular cell proliferation, and migration *in vitro*. It helps to maximize the continuous hypoxic pulmonary vasoconstriction response and PA remodeling in hypoxia-induced PH and other forms of PH (47, 48). ARHGEF1 (Rho guanine nucleotide exchange factor 1) is related to cytoskeleton structure, microtubule, and the actin cytoskeleton. Recently, it has been described as RhoA-specific nucleotide exchange factor. It catalyzes the exchange of GDP and GTP to produce activated RhoA and participates in the regulation of RhoA activity (49, 50). Tong et al. used the gene expression profile GSE22356 to develop early biomarkers to distinguish systemic sclerosis with or without PH from the normal population using bioinformatics approaches. They found that *TNFAIP6* (TNF-α-stimulated gene-6) as a hub gene showed an obvious positive correlation between systemic sclerosis with PH (51). Miao et al. (52) also used the gene expression profile GSE130391 to identify five key immune cell-related DEGs, including *PFN2* (profilin 2), in the PA of patients with CTEPH.

*PLOD2* (53)*, FSCN1* (54), and *NR2F2* (55) were suggested to play an essential role in the normal development of lung and pulmonary vessels. In addition to these reported genes, six upregulated genes (*WASF1, RB1, ITM2B, ADD3, APAF1*, and *LIMD1*) and eight downregulated candidate genes (*DAPK1, PKN1, TRIP10, DRAM1, ULK2, DMPK, LPR12*, and *MYOF*) in PH have not been reported yet.

Some of those genes were randomly selected for verification in two types of rat PH models. Our qRT-PCR results indicated that Wiskott–Aldrich syndrome protein-family 1 (*WASF1)* and *RB1* are significantly upregulated in rat PAH models. The downstream effector molecule, *WASF1*, is implicated in the transmission of signals from small GTPases and tyrosine kinase receptors to the actin cytoskeleton; it also enhances the actin filaments formation (56) and is an important pro-autophagic protein capable of augmenting cell survival (57). *LIMD1* is widely expressed in the lungs, according to Fagerberg et al. (58); this gene encodes an adapter or scaffold protein called LIM domain-containing protein 1 that is involved in the formation of a large number of protein complexes and multiple cellular functions, including gene transcription suppression, cytoskeletal structure, cell fate determination, cell–cell adhesion, cell differentiation, migration, and proliferation (59–64). Furthermore, LIMD1 functions as a hypoxic regulator by connecting the gap between prolyl hydroxylases and VHL, allowing for effective hypoxia-induced factor 1A (HIF1A) degradation (60). When phosphorylated by CDK3/cyclin-C, *RB1* is a major regulator of cell division and works as a tumor suppressor, promoting the G0-G1 transition. LIMD1 binds to phosphorylated RB1, interacts with E2F1, and inhibits E2F1's transcriptional activity, causing cell cycle arrest (64). Myoferlin (MYOF) is a calcium/phospholipid-binding protein involved in muscle growth and repair (65, 66). MYOF functions in migration, proliferation, and nitric oxide (NO) release, which occurs in response to vascular endothelial growth factor (VEGF) in endothelial cells (67, 68). According to the function of these genes, we can speculate that they may be involved in vascular cell proliferation, migration, and vascular remodeling.

qPCR results showed that mRNA expression of some DEGs was consistent with mRNA-Seq, and the expression trend of *IL1R1, DAPK1*, and *TNFAIP6* genes in animal models was inconsistent with the mRNA-Seq results. One possible reason is that the materials used for data analysis and model validation come from two different species. The materials used in the sequencing database are PAs from patients with PH, and the material used for PCR verification are PAs from the PH rat model. Although the expression profiles of some genes are similar in two different species, some other genes may have different expression trends. In addition, due to the complex pathogenesis of PH, the gene expression patterns of some genes may be different in different individuals. Evidence showed that the mRNA expression of *IL1R1* (44) and *TNFAIP6* (69) are upregulated in patients with IPAH by validation from qPCR. consistently, *IL1R1* (44) is upregulated in monocrotaline-induced PH rats. These results are consistent with our qPCR results from hypoxia with Sugen-induced PH rats. Western blot analysis showed that ARHGEF1 and RB1 are decreased in hypoxia with Sugen-induced PH rats. Their protein expression levels are opposite to the gene expression levels, possibly due to post-translational modifications such as degradation by ubiquitination.

Pathway analysis revealed that the NF-κB signaling pathway, osteoclast differentiation, and the regulation of cytokine–cytokine receptor interaction were their common pathways. These functional features and pathways have been widely confirmed in different PH, suggesting that these genes are involved in PA remodeling in PH. From the function and research results of these genes, we can speculate that they must be related to PA remodeling. Thus, due to their key roles in the PA, alterations in their expression may contribute to the development of IPAH and CTEPH.

Although different PH groups share PA remodeling-associated overlapping genes, each group of PH disease has specific pathogenesis. In 2020, Halliday et al. compared the morphology of human PA among CTEPH and IPAH and the control group and found significant differences among them. The expression of all markers in PAECs and PSMCs of CTEPH was generally lower (18). They speculate that it may be caused by cell dedifferentiation (18). In this study, the non-overlapping DEGs may represent the key factors in the different pathogenesis of CTEPH and IPAH.

## Conclusion

This study identified the DEGs and 10 hub genes of IPAH and CTEPH, and 22 hub genes of 120 common DEGs in IPAH and CTEPH using bioinformatics analysis. These results provide the most up-to-date information regarding mechanism research and potential treatment strategies for PA remodeling-related diseases, such as IPAH, CTEPH, and related diseases. However, this study has a limitation, that is, our data could not be interpreted with regard to age, sex, or race due to insufficient sample size. Nevertheless, this study will provide some new target genes for further study of PH mechanisms and treatment.

## Data availability statement

The original contributions presented in the study are included in the article/supplementary material, further inquiries can be directed to the corresponding author/s.

## Ethics statement

The animal study was reviewed and approved by Huazhong University of Science and Technology (HUST) Animal Care and Use Committee, and conducted following the Guidelines for the Care and Use of Laboratory Animals (S1821).

## Author contributions

Conceptualization and experimental design: RT. Experiments, data collection and analysis, and review and editing: RT and QY. Experiments, data analysis, and editing: DY, CX, JC, and TZ. Original draft preparation and data analysis: RT and JA-A. All authors contributed to the article and approved the submitted version.

## Funding

This research was funded by the National Natural Science Foundation of China (81700055), the Outstanding Talent Research Funding of Xuzhou Medical University (Grant No. D2016021), the Natural Science Foundation of Jiangsu Province (BK20160229), and the Natural Science Foundation of the Jiangsu Higher Education Institutions of China (18KJB310017).

## Conflict of interest

The authors declare that the research was conducted in the absence of any commercial or financial relationships that could be construed as a potential conflict of interest.

## Publisher's note

All claims expressed in this article are solely those of the authors and do not necessarily represent those of their affiliated organizations, or those of the publisher, the editors and the reviewers. Any product that may be evaluated in this article, or claim that may be made by its manufacturer, is not guaranteed or endorsed by the publisher.
